# Supplementary material for: A Novel m7G-Related Gene Signature Predicts the Prognosis of Colon Cancer
Source: Cancers (Basel). 2022 Nov 10;14(22):5527. doi: 10.3390/cancers14225527 (PMC9688272; doi:10.3390/cancers14225527)
Supplement: Supplementary file 1 [file cancers-14-05527-s001.zip › cancers-1992688-supplementary figures.pdf]

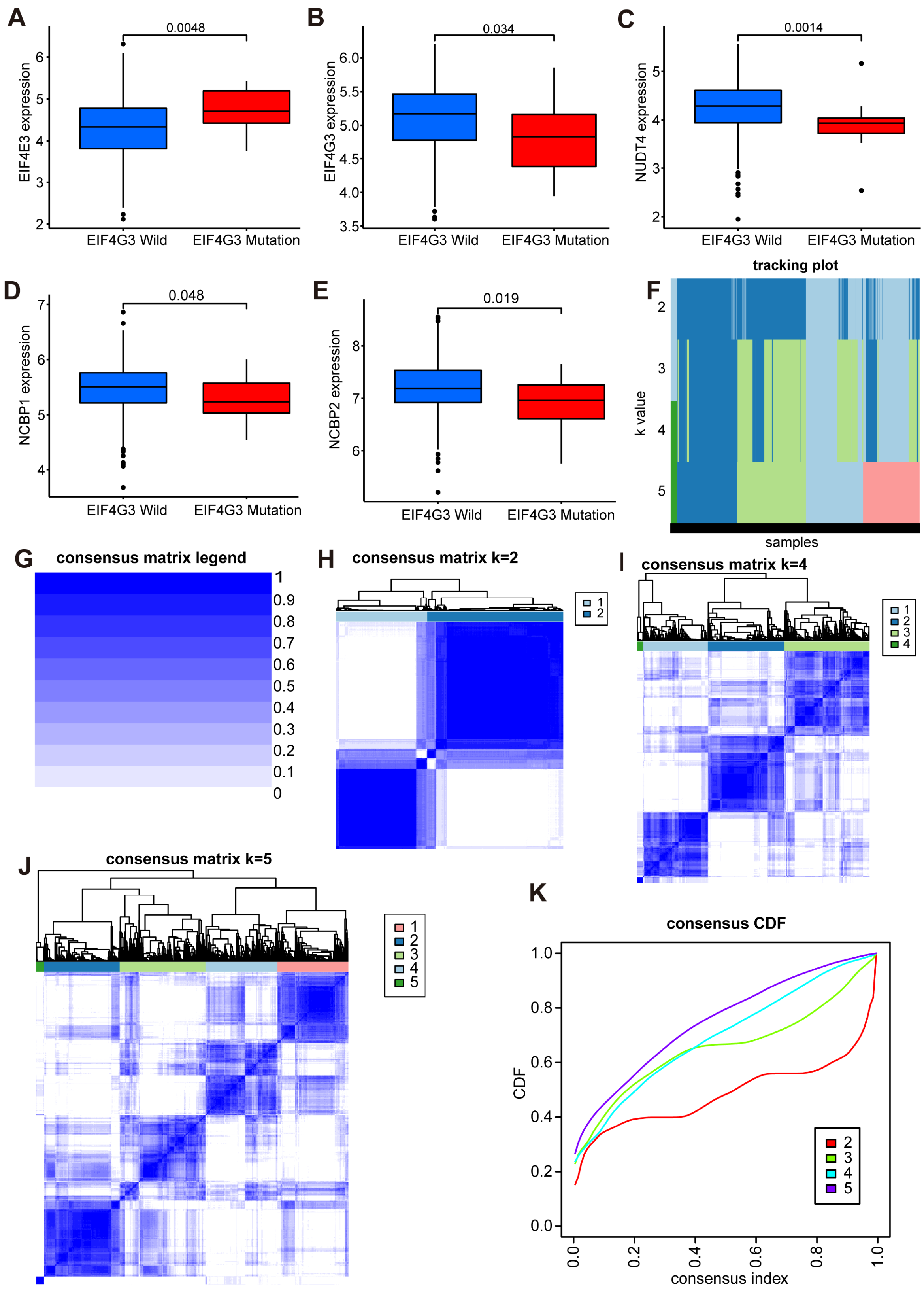

Figure S1. Correlation analysis and construction of m7G clusters

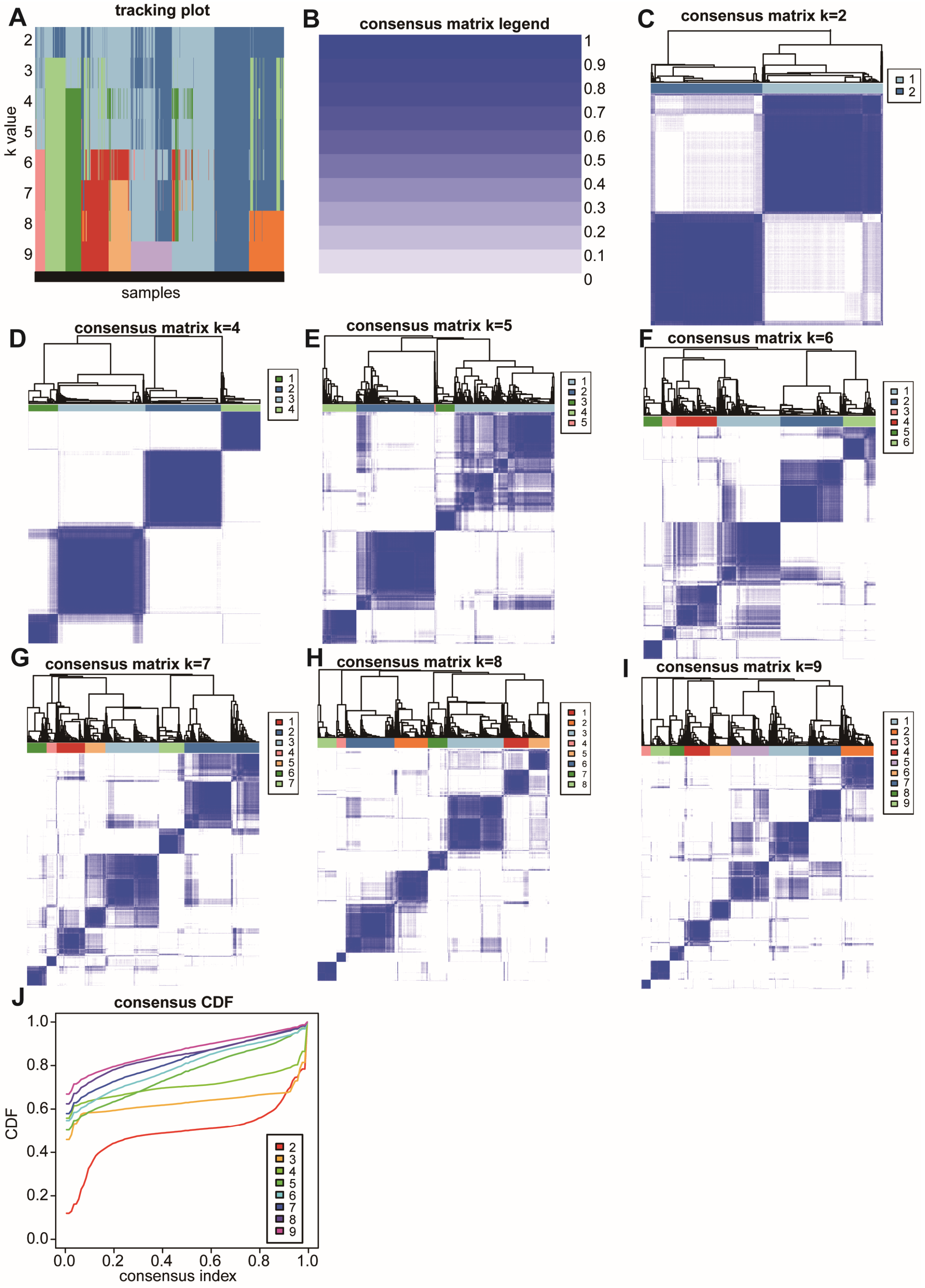

Figure S2. Gene clustering based on prognostic m7G-related DEGs in CC patients.

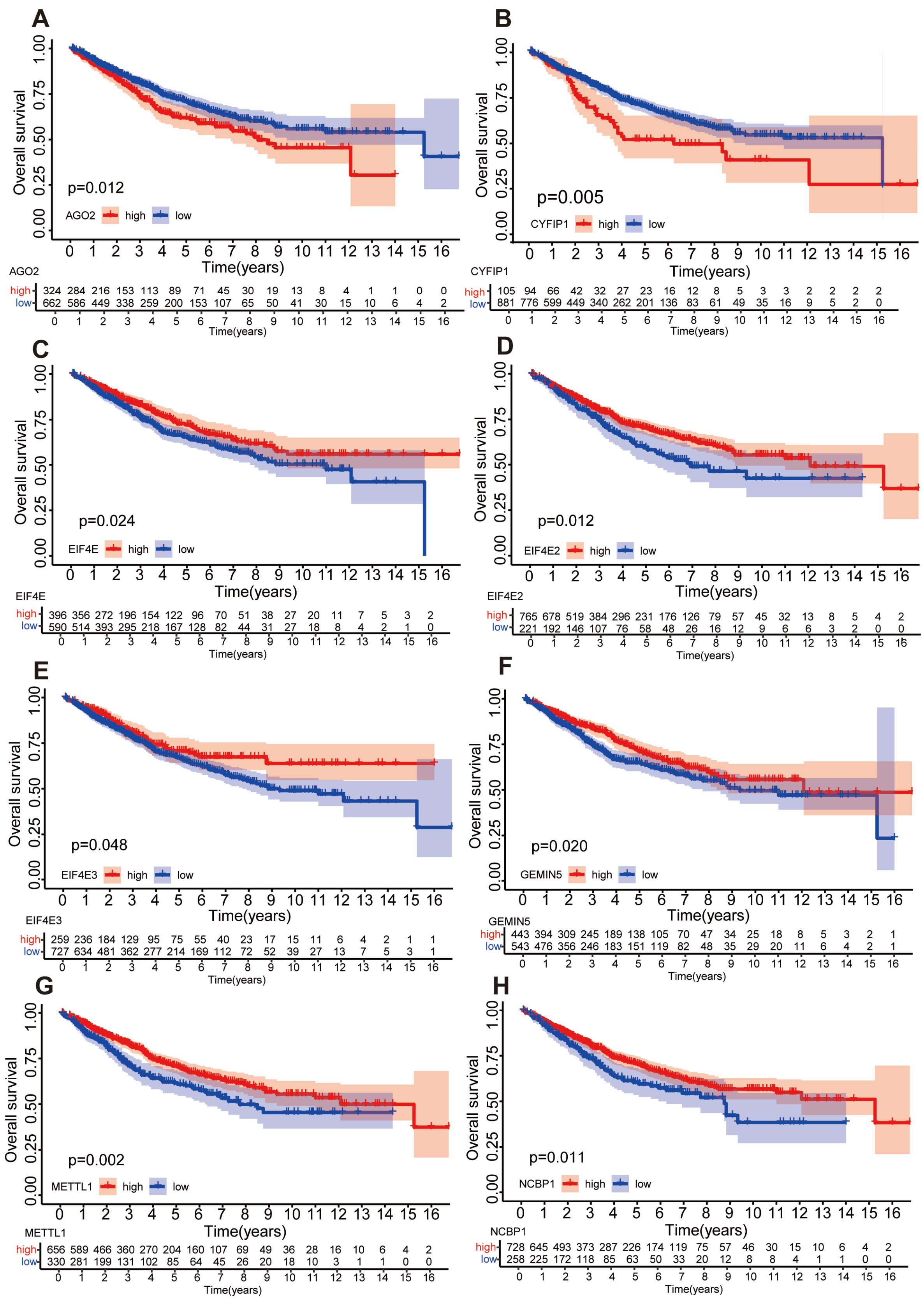
A
p=0.012
AGO2 high low
Overall survival
Time(years)
AGO2
high 324 284 216 153 113 89 71 45 30 19 13 8 4 1 1 0 0
low 662 586 449 338 259 200 153 107 65 50 41 30 15 10 6 4 2
B
p=0.005
CYFIP1 high low
CYFIP1
high 105 94 66 42 32 27 23 16 12 8 5 3 3 2 2 2 2
low 881 776 599 449 340 262 201 136 83 61 49 35 16 9 5 2 0
C
p=0.024
EIF4E high low
EIF4E
high 396 356 272 196 154 122 96 70 51 38 27 20 11 7 5 3 2
low 590 514 393 295 218 167 128 82 44 31 27 18 8 4 2 1 0
D
p=0.012
EIF4E2 high low
EIF4E2
high 765 678 519 384 296 231 176 126 79 57 45 32 13 8 5 4 2
low 221 192 146 107 76 58 48 26 16 12 9 6 6 3 2 0 0
E
p=0.048
EIF4E3 high low
EIF4E3
high 259 236 184 129 95 75 55 40 23 17 15 11 6 4 2 1 1
low 727 634 481 362 277 214 169 112 72 52 39 27 13 7 5 3 1
F
p=0.020
GEMIN5 high low
GEMIN5
high 443 394 309 245 189 138 105 70 47 34 25 18 8 5 3 2 1
low 543 476 356 246 183 151 119 82 48 35 29 20 11 6 4 2 1
G
p=0.002
METTL1 high low
METTL1
high 656 589 466 360 270 204 160 107 69 49 36 28 16 10 6 4 2
low 330 281 199 131 102 85 64 45 26 20 18 10 3 1 1 0 0
H
p=0.011
NCBP1 high low
NCBP1
high 728 645 493 373 287 226 174 119 75 57 46 30 15 10 6 4 2
low 258 225 172 118 85 63 50 33 20 12 8 8 4 1 1 0 0

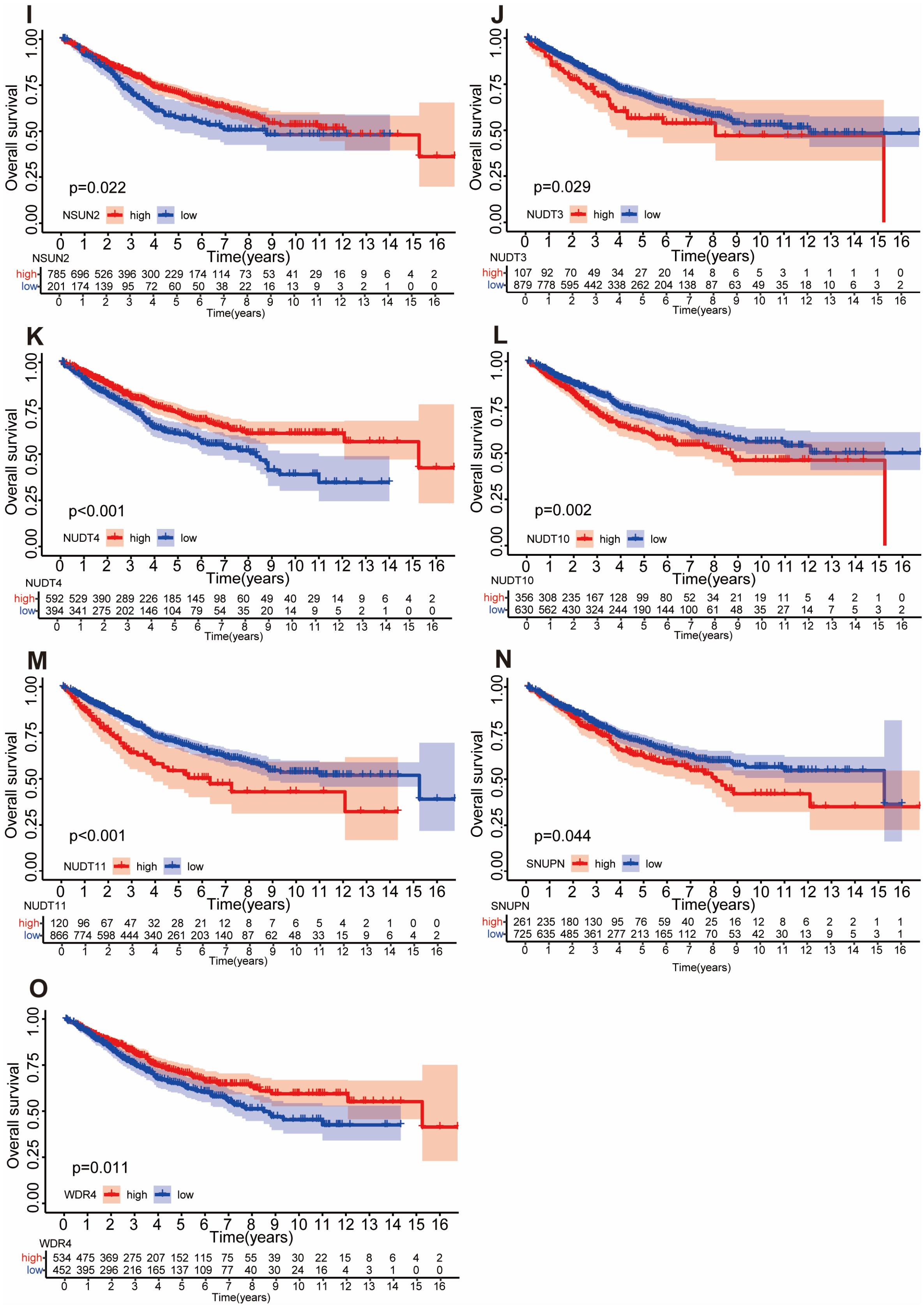

Figure S3. The KM Curves of OS of CC Patients with High and Low m7G Gene Expression
